# Microglial activation in early Alzheimer trajectory is associated with higher gray matter volume

**DOI:** 10.1212/WNL.0000000000007133

**Published:** 2019-03-19

**Authors:** Grazia Daniela Femminella, Melanie Dani, Melanie Wood, Zhen Fan, Valeria Calsolaro, Rebecca Atkinson, Trudi Edginton, Rainer Hinz, David J. Brooks, Paul Edison

**Affiliations:** From the Department of Medicine (G.D.F., M.D., M.W., Z.F., V.C., R.A., D.J.B., P.E.), Imperial College London; Department of Psychology (T.E.), University of London, London; Wolfson Molecular Imaging Centre (R.H.), University of Manchester, UK; and Department of Nuclear Medicine (D.J.B.), Aarhus University, Denmark.

## Abstract

**Objective:**

To investigate the influence of microglial activation in the early stages of Alzheimer's disease trajectory, we assessed the relationship between microglial activation and gray matter volume and hippocampal volume in patients with mild cognitive impairment (MCI).

**Methods:**

In this study, 55 participants (37 with early stages of MCI and 18 controls) underwent [^11^C]PBR28 PET, a marker of microglial activation; volumetric MRI to evaluate gray matter and hippocampal volumes as well as clinical and neuropsychometric evaluation. [^11^C]PBR28 V_T_ (volume of distribution) was calculated using arterial input function and Logan graphical analysis. Gray matter volume and hippocampal volumes were calculated from MRI for each participant. Statistical parametric mapping software was used to perform voxel-wise correlations and biological parametric mapping analysis. Amyloid status was assessed using [^18^F]flutemetamol PET.

**Results:**

Higher [^11^C]PBR28 V_T_ in different cortical areas correlated with higher gray matter volume in both amyloid-positive and -negative MCI. In addition, higher hippocampal volume correlated with higher cortical [^11^C]PBR28 Logan V_T_.

**Conclusions:**

In this in vivo study, we have demonstrated that microglial activation quantified using [^11^C]PBR28 PET was associated with higher gray matter volume and higher hippocampal volume in patients with MCI. This might suggest that microglial activation may not always be associated with neuronal damage, and indeed it may have a beneficial effect in the early stages of the Alzheimer trajectory. While further longitudinal studies are necessary, these findings have significant implications on therapeutic strategies targeting microglial activation.

Amyloid plaque formation and tau aggregation are the hallmarks of Alzheimer disease (AD) pathology. The failure of antiamyloid strategies in AD and mild cognitive impairment (MCI)^1^ suggests that other mechanisms, such as neuroinflammation, may have a significant role in the neurodegenerative process.^2,3^ Microglia are responsible for maintaining the homeostasis and immune defense mechanism in brain, and become activated by any brain insults. Both β-amyloid and tau can trigger microglia activation,^4–7^ contributing to neuronal damage in established AD. In prodromal AD, microglial activation might have a protective role by clearing amyloid.^2,8,9^ Microglial activation is characterized by upregulation of the mitochondrial translocator protein, TSPO.^10,11^ Studies using the microglial marker [^11^C](R)PK11195-PET have demonstrated increased microglial activation in late AD, which correlated inversely with memory performance.^12^ However, the role of microglial activation in the early stages of neurodegenerative diseases is still debated. Recently, we proposed that there could be 2 peaks of microglial activation in the AD trajectory, and indeed, the early peak could be protective while the later peak could be detrimental.^26^

[^11^C]PBR28 is a TSPO ligand with 80-fold-higher affinity^13^ than [^11^C]PK11195, and minimal affinity for astrocytes.^14^ Gray matter volume measures the amount of gray matter within a region relative to other tissue types.^15^ It relates to the amount of atrophy, providing information on brain volume loss.^15^ In this study, we evaluated whether microglial activation in MCI assessed using [^11^C]PBR28 PET is associated with brain volume preservation, measured by gray matter and hippocampal volumes.

## Methods

### Study population

Fifty-five participants (37 patients with MCI and 18 healthy controls [HCs]) were recruited from the Imperial College memory clinics, dementia registry, and memory clinics around London. Patients with MCI had a clinical diagnosis made by a specialist consultant at memory clinics, and the study investigators reviewed the patient according to the Petersen^16^ and National Institute on Aging–Alzheimer’s Association criteria.^17^ Objective memory loss was measured by education-adjusted scores on the Wechsler Memory Scale–Logical Memory. The following were other inclusion criteria: (1) age range 50–85 years; (2) ability to give informed consent; and (3) at least 8 years of education. Exclusion criteria included the following: (1) depression; (2) any significant disease or unstable medical condition that could influence neuropsychological testing; (3) contraindications to MRI; (4) history of schizophrenia, schizoaffective disorder, bipolar disorder, or any history of electroconvulsive therapy; and (5) significant white matter microvascular disease on MRI scans. All participants had a detailed medical and neurologic workup and detailed neuropsychometric testing, which included the National Adult Reading Test, the Rey-Osterrieth Complex Figure, the Wechsler Memory Scale–Logical Memory, the Hopkins Verbal Learning Test–Revised, semantic and verbal fluency tests, Digit Span, Letter-Number Sequencing, and Trail Making A and B tests. All participants underwent genetic testing for the presence of Ala147Thr polymorphisms of their *TSPO* genes to establish their binding status for [^11^C]PBR28: they were stratified into high-affinity binders (HABs), mixed-affinity binders (MABs), or low-affinity binders.^18^ Because low-affinity binders show negligible binding, they were excluded from the study.

### Standard protocol approvals, registrations, and patient consents

This study was approved by the local and regional research ethics committee (London Riverside Research Ethics Committee–National Health Research Services, Health Research Authority, UK), and approval for the administration of radioactivity was given by ARSAC (Administration of Radioactive Substances Advisory Committee).

### [^11^C]PBR28 PET, [^18^F]flutemetamol PET, and MRI acquisition and analysis

#### [^11^C]PBR28 PET

All participants had [^11^C]PBR28 PET scans using a Siemens Biograph TruePoint PET/CT scanner (Siemens, Erlangen, Germany) at Imanova, London. [^11^C]PBR28 was manufactured and supplied by Imanova, London. A low-dose CT scan was initially acquired for attenuation correction and to position the patient. A mean activity of 300.3 (±37) MBq [^11^C]PBR28 in 20 mL normal saline was injected IV over 20 seconds. Three-dimensional dynamic data were acquired in list mode, and reconstruction was performed using standard Siemens software. Arterial whole blood activity was measured continuously with an online detector for the first 15 minutes while discrete blood samples were taken at 5, 10, 20, 30, 50, 70, and 90 minutes and centrifuged to detect whole blood and plasma radioactivity and to measure radioactive metabolite levels. [^11^C]PBR28 dynamic PET was corrected for head motion using the frame-by-frame realignment tool in statistical parametric mapping (SPM12, Wellcome Trust Centre for Neuroimaging) software. In this study, we have used Logan graphic analysis to generate the parametric maps for [^11^C]PBR28.^19,20^ The dynamic data of [^11^C]PBR28 tracer activity, plasma input function, and brain tissue were converted into a linear plot using graphic analysis by the MICK program, developed in MATLAB. Logan parametric maps of V_T_ (volume of distribution) were then generated using MICK parametric mapping software.

#### Creation of object map

Using SPM12 and Analyze11, an individualized object map in PET space was created for each participant using the following steps: (1) the individual's MRI was coregistered to their native PET space; (2) gray matter, white matter, and CSF maps were generated by segmentation of the coregistered MRI, and a binarized gray matter mask was created using a threshold of 0.5; (3) a probabilistic region-of-interest (ROI) atlas^21^ in MNI (Montreal Neurological Institute) space was transformed into native PET space; and (4) the probabilistic atlas in PET space was then applied to the binarized individual gray matter mask to generate individualized gray matter ROIs. Regional Logan V_T_ was estimated for frontal, temporal, parietal, and occipital cortical regions by sampling individual parametric maps. Additional ROIs in AD, such as posterior cingulate, anterior cingulate, and medial temporal lobe, were also sampled.

#### MRI scans

MRI scans were acquired on a Verio, 3T clinical MRI system (Siemens, VB19) using a 32-channel head coil, and included a T1-weighted magnetization-prepared rapid–acquisition gradient echo sequence (repetition time = 2,400 milliseconds [ms], echo time = 3.06 ms, flip angle of 9, inversion time = 900 ms, matrix = 256 × 256) generating 1 mm^3^ isotropic voxels, for coregistration with the PET images for regional PET analysis. T2-weighted MRI sequences were also acquired to evaluate vascular and other structural abnormalities. The hippocampal volume was calculated using FreeSurfer (Harvard Medical School; surfer.nmr.mgh.harvard.edu).

#### Gray matter volume

**Figure 1:**
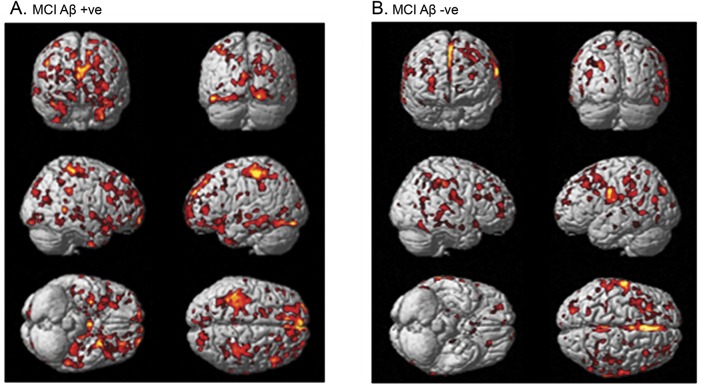
Biological parametric mapping correlation between [^11^C]PBR28 volume of distribution and gray matter volume Correlation between higher [^11^C]PBR28 and higher MRI gray matter density in (A) amyloid-positive patients with MCI and (B) amyloid-negative patients with MCI. Aβ = β-amyloid; MCI = mild cognitive impairment.

Preprocessing was applied to generate a modulated normalized gray matter MRI map using voxel-based morphometry (VBM) for each participant.^15,22^ Initially, the raw MRIs were segmented into gray matter, white matter, and CSF. The gray matter was then normalized, and the gray matter–normalized parameters were further used in the spatial normalization of the raw whole brain image. Modulation was applied to correct for the global confounding effects during the spatial normalization, in order to account for the absolute volume of the gray matter (figure 1).^15^

#### [^18^F]Flutemetamol PET

[^18^F]Flutemetamol was manufactured and supplied by GE Healthcare (Waukesha, WI) and PET scans were performed at the Imperial College Clinical Imaging Facility using a Siemens Biograph 6 PET/CT scanner. [^18^F]Flutemetamol was injected IV as a single bolus over 20 seconds into an antecubital vein. Mean injected activity was 182.3 (±2.5) MBq. Fifty-two participants (17 HCs and 35 patients with MCI) had [^18^F]flutemetamol PET scans. Three participants were not available to complete the [^18^F]flutemetamol PET scans within the window allowed by the study protocol. The data were acquired 90 to 120 minutes post tracer injection using listmode in 3D. After creating an object map as described above, [^18^F]flutemetamol uptake RATIO images in PET space was created using cerebellar gray matter as reference, and RATIO images were then sampled. To determine whether [^18^F]flutemetamol uptake was abnormally increased in patients with MCI at the ROI level, we compared individual regional values to the control mean of the predefined regions. We considered a participant to be amyloid positive if the uptake ratio was >2 SD above the HC mean in at least one of our predefined cortical ROIs (frontal, temporal, parietal, and occipital cortices, anterior and posterior cingulate gyri).^23^

### Single-subject analysis

Because a classic ROI approach for interrogating inhomogeneous microglial activation tends to underestimate the localized voxel-wise small changes within any predefined ROI region, we developed individualized SPM maps by comparing each patient against HCs to localize significant clusters of binding change.^24,25^ VOI (voxels of interest) maps for increased [^11^C]PBR28 V_T_ for each participant were generated by extracting the significant clusters from the single-subject result in SPM12. The volume of each pathologic process was calculated using Analyze11,^27^ where each patient's [^11^C]PBR28 PET image was compared with their corresponding HCs for the HAB or MAB cohort. The statistical threshold for significance was set at *p* < 0.05 and an extent threshold of 50 voxels.

### Correlation between [^11^C]PBR28 and hippocampal volume

Initially, [^11^C]PBR28 Logan parametric maps for individual participants were coregistered to the MRI, and then spatially transformed into MNI space. To evaluate the voxel-wise correlation between microglial activation and hippocampal volume, multiple regression was performed in SPM12 using Logan parametric maps of [^11^C]PBR28 V_T_ as the dependent variable and hippocampal volumes calculated using FreeSurfer as the covariate of interest.

### Generation of *z* score maps and biological parametric mapping correlation, and statistical analysis

To compare the voxel-wise correlation between [^11^C]PBR28 V_T_ with the gray matter volume in patients with MCI, *z* score maps were generated for each participant for each modality. Smoothed and normalized individual Logan parametric maps of [^11^C]PBR28 V_T_ were used for localizing microglial activation. The individual *z* score maps for [^11^C]PBR28 V_T_ and modulated gray matter map were created in SPM12 using the following formulae:
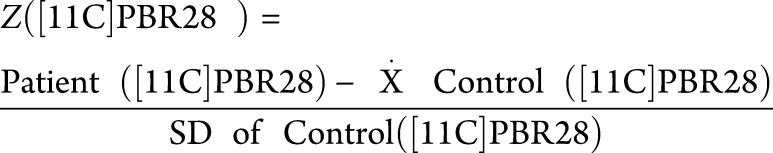

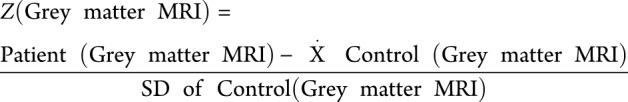
where Z denotes *z* score map, Ẋ denotes mean, and SD denotes standard deviation. Then individual *z* score maps were applied in the biological parametric mapping (BPM) toolbox to assess the voxel-wise correlation, which uses the general linear model to perform regressions to provide a sophisticated comparison between different imaging modalities at a voxel level, as shown in figure 1.^27^ Individual *z* score MRI maps provide the whole-brain profile of degree of gray matter volume. The *z* score maps for HAB and MAB were created separately using corresponding controls. HAB and MAB *z* score maps were combined for the BPM analysis. Similarly, individual *z* score maps were created for [^18^F]flutemetamol, and BPM correlation between microglial activation and amyloid pathology was evaluated in amyloid-positive patients.^28^

Statistical analysis was performed using IBM SPSS 22 in Windows 7 (IBM Corp., Armonk, NY). Continuous variables were expressed as mean ± SD. The Student *t* test was used to compare normally distributed continuous variables between the 2 groups. Categorical variables were compared with the χ^2^ statistic. Significance was set at a threshold of *p* < 0.05.

### Data availability

These data may be shared with qualified scientific and medical researchers, upon researcher's request, as necessary for conducting research depending on the terms of our regulatory approvals and institutional policy.

## Results

Patient demographics and neuropsychometric tests scores are presented in table 1, while volumetric MRI measures are presented in table 2.

**Table 1:**
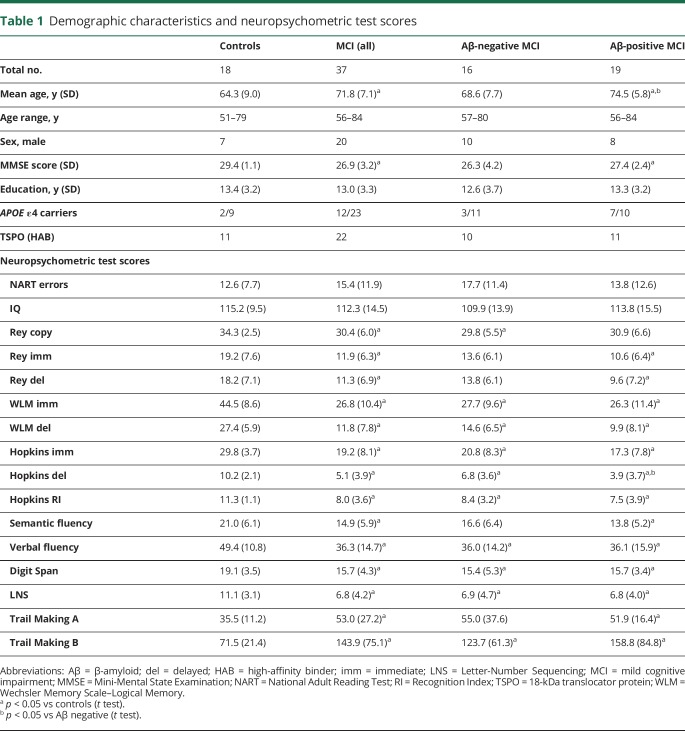
Demographic characteristics and neuropsychometric test scores

**Table 2:**
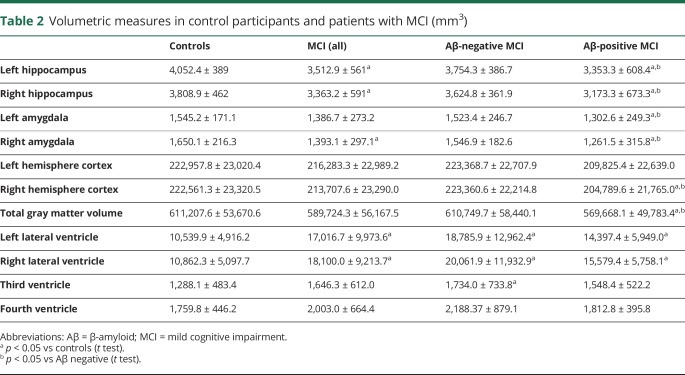
Volumetric measures in control participants and patients with MCI (mm^3^)

### Voxel-wise correlation between [^11^C]PBR28 V_T_, hippocampal volume, and gray matter volume

**Figure 2:**
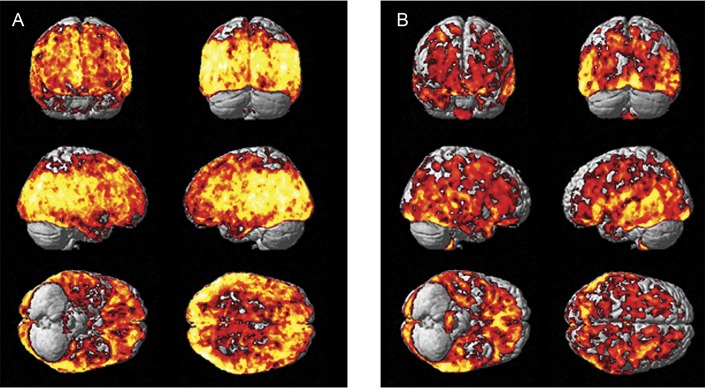
Biological parametric mapping correlation between [^11^C]PBR28 V_T_ and hippocampal volume Correlation between higher microglial activation ([^11^C]PBR28 V_T_) and hippocampal volume in patients with HAB (A) and MAB (B) status and mild cognitive impairment. V_T_ = volume of distribution.

The voxel-wise BPM correlation analysis demonstrated correlation between higher [^11^C]PBR28 Logan V_T_ and higher MRI gray matter volume at a voxel threshold of *p* < 0.05 with an extent threshold of 50 voxels. Separating the patients with MCI into amyloid-positive (n = 19) and -negative (n = 16) subgroups, the voxel-wise BPM correlations showed correlations between gray matter volume and [^11^C]PBR28 V_T_ in both subgroups, mainly evident in frontal, temporal, and parietal regions in amyloid-positive patients with MCI (figure 2A, table 3, and data available from Dryad, supplemental table 3, doi.org/10.5061/dryad.6mp3r21) and frontal and parietal regions in amyloid-negative patients (figure 2B, table 4). The BPM analysis between microglial activation and amyloid pathology in amyloid-positive patients with MCI showed areas of correlation in the posterior cingulate areas and middle frontal gyrus (data available from Dryad, supplemental table 4, doi.org/10.5061/dryad.6mp3r21).

**Table 3:**
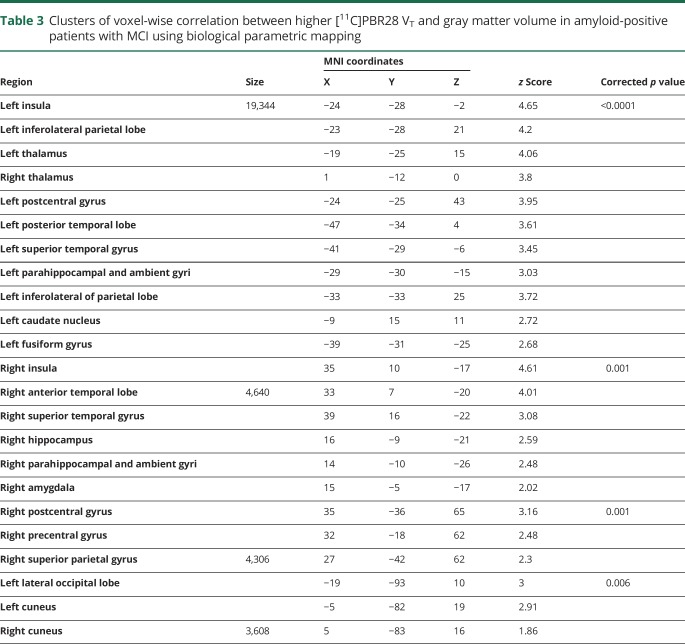
Clusters of voxel-wise correlation between higher [^11^C]PBR28 V_T_ and gray matter volume in amyloid-positive patients with MCI using biological parametric mapping

**Table 4:**
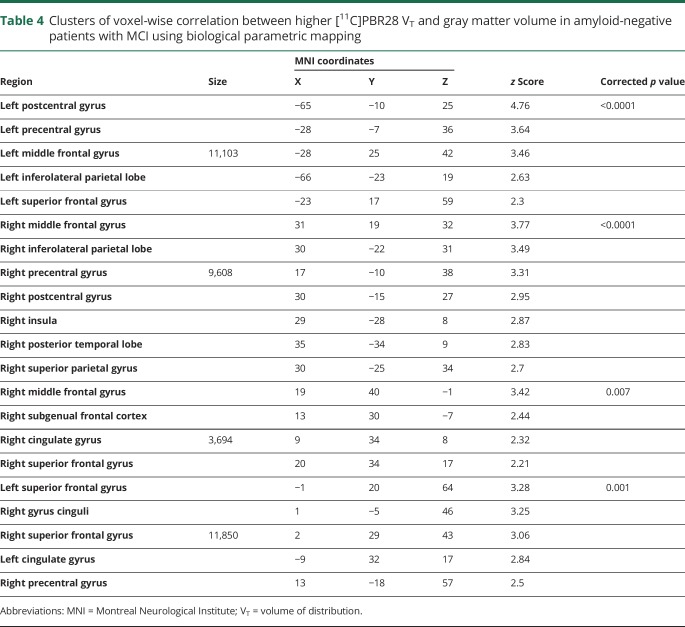
Clusters of voxel-wise correlation between higher [^11^C]PBR28 V_T_ and gray matter volume in amyloid-negative patients with MCI using biological parametric mapping

**Figure 3:**
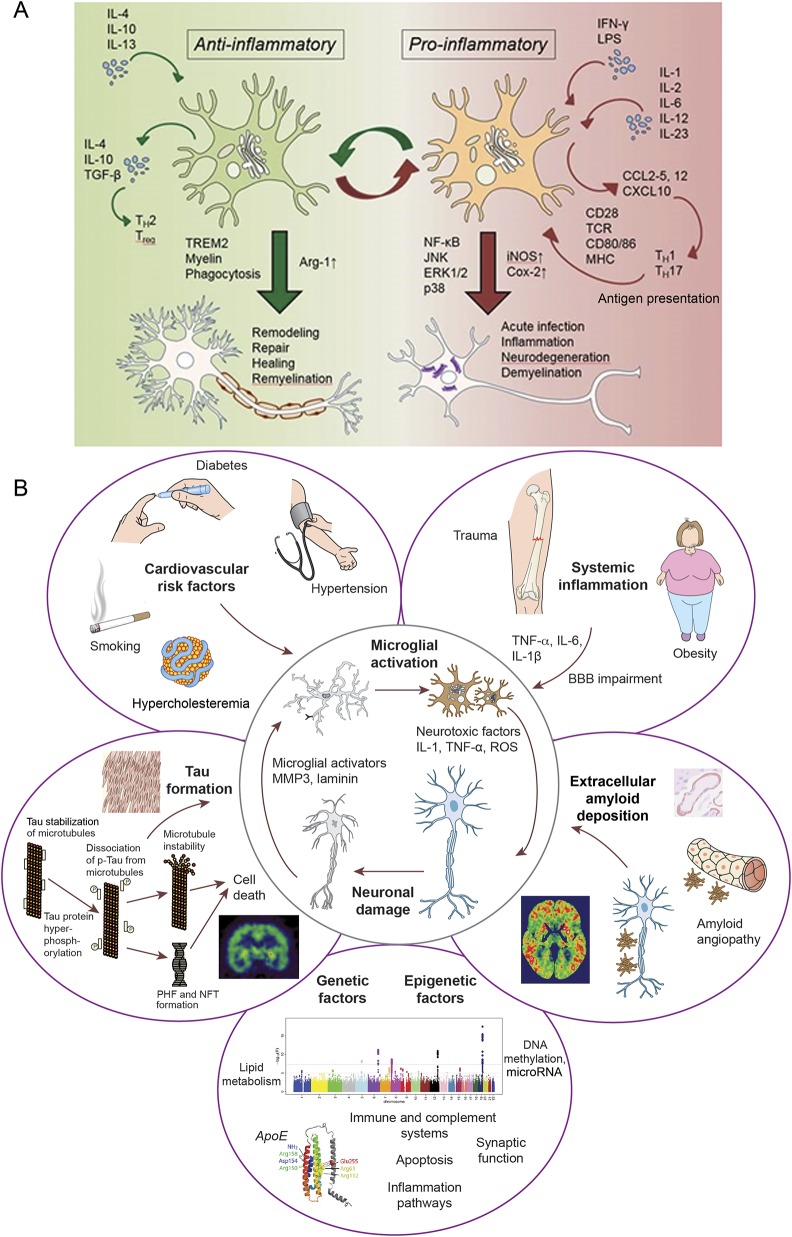
Probable mechanism of microglia activation and factors contributing to neuroinflammation (A) Microglia can be activated by either anti-inflammatory stimuli (IL-4, IL-10, or IL-13) or proinflammatory cytokines (IFN-, LPS) that determine the polarization status of the cell. The anti- and proinflammatory responses involve the activation of different intracellular pathways and result in opposite effects on neuronal cells. (B) Systemic (cardiovascular risk factors and systemic inflammation), local (amyloid deposition and tangle formation), and genetic factors contribute to microglial activation. BBB = blood-brain barrier; COX-2 = cyclooxygenase-2; IFN = interferon; IL = interleukin; iNOS = inducible nitric oxide synthase; LPS = lipopolysaccharide; MHC = major histocompatibility complex; NF-κB = nuclear factor κB; NFT = neurofibrillary tangles; PHF = paired helical filaments; p-tau = phosphorylated tau; ROS = reactive oxygen species; TCR = T cell receptor; TGF-β = transforming growth factor β.

The mean total hippocampal volume was lower in patients with MCI (left 3,512.9 ± 561 mm^3^; right 3,363.2 ± 591 mm^3^) (*p* < 0.05) compared to the HCs (left 4,052.4 ± 389 mm^3^; right 3,808.9 ± 462 mm^3^). The multiple regression analysis in SPM revealed a correlation between higher [^11^C]PBR28 V_T_ and total higher hippocampal volume in the MCI cohort using binding status (high or mixed affinity) as a covariate. Evaluating the participants separately depending on their TSPO binding status, in the MCI cohort with HABs for TSPO, higher hippocampal volume correlated with higher [^11^C]PBR28 V_T_ in frontal, temporal, parietal, and occipital lobes (voxel threshold of *p* < 5e-11 and extent threshold of 200 voxels) (figure 3A, table 5). In the MCI cohort with MABs for TSPO, higher hippocampal volume was correlated with higher [^11^C]PBR28 V_T_ in temporal and occipital lobes (voxel threshold of *p* < 5e-7, and extent threshold of 200 voxels) (figure 3B, table 5). Regional [^11^C]PBR28 V_T_ in controls and patients with MCI are given in data available from Dryad (supplemental table 1, doi.org/10.5061/dryad.6mp3r21), and regional [^18^F]flutemetamol target-to-cerebellum ratios are presented in data available from Dryad (supplemental table 2, doi.org/10.5061/dryad.6mp3r21). Individually, 13 of 37 patients with MCI (35%) were classified as having increased [^11^C]PBR28 V_T_ compared to controls on single-subject SPM analysis. At the ROI level, there was no correlation between higher [^11^C]PBR28 V_T_ and higher gray matter or hippocampal volume.

**Table 5:**
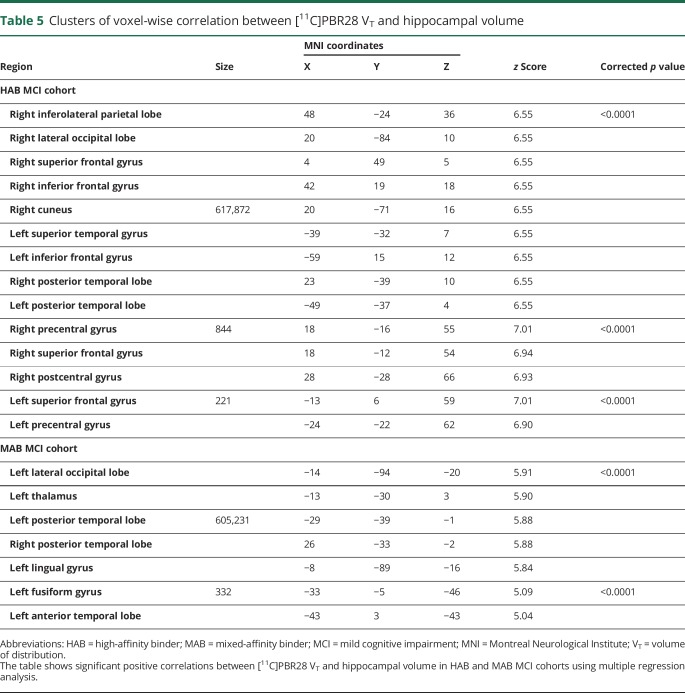
Clusters of voxel-wise correlation between [^11^C]PBR28 V_T_ and hippocampal volume

## Discussion

In this study, we have demonstrated that [^11^C]PBR28 Logan V_T_ in different cortical areas is associated with higher hippocampal volume and greater gray matter volume in the early stages of the AD trajectory. This may suggest that microglia, which maintains the natural homeostatic/defense mechanism of the brain, initially responds by trying to protect the brain. Recent studies have demonstrated that [^11^C]PBR28 is specific for activated microglia,^14,29^ and this study highlights the role of microglia in protecting the brain in the very early stages of neurodegeneration. This is in contrast to the role of microglia in established AD, where microglial activation exerts a deleterious effect.

Because microglial cells are the primary immune effector cells in the brain, in response to any kind of brain damage, they become activated and undergo morphologic and functional transformations, along with structural changes. Microglia express (1) AMPA (α-amino-3-hydroxy-5-methyl-4-isoxazole propionic acid) receptors to respond to glutamate, (2) P2 purinoceptors to respond to ATP (adenosine 5′-triphosphate) and other nucleotides, (3) cytokine receptors to respond to tumor necrosis factor α and interleukin 1β, and (4) receptors for the major histocompatibility complex class antigens and chemokines. Activation of these receptors induces signaling cascades that are involved in microglia chemotaxis, activation, and phagocytosis, and protecting the brain from injury.^2^ It is suggested that amyloid aggregation can induce microglial activation, which may have a protective effect by promoting oligomer and fibril clearance in the early phases of the disease.^30^ One could argue the preservation of the brain volume associated with increased microglial activation seen in this study is consistent with the natural defense mechanism of the microglial response to neuronal injury; indeed, the participants included in this study have predominantly early MCI, in which their impairment in cognitive function is minimal.

In our cohort, 55% of all participants with MCI had a positive amyloid scan, and therefore, according to the recently revised research criteria for a biological definition of AD, fall within the trajectory of the AD continuum.^31^ Although their “T” (aggregated tau) status is unknown, they are “A” (amyloid) positive and “N” (neurodegeneration) positive as a group, based on the ATN classification. The other 45% of patients with MCI have a negative amyloid scan; therefore, they can be classified as “non-AD pathologic change” or “suspected non-AD pathophysiology.” This latter group might include participants with primary age-related tauopathy, hippocampal sclerosis, TDP-43 (TAR DNA-binding protein 43) pathology, primary progressive aphasia, or cerebrovascular disease.^32^ In both amyloid- and nonamyloid-driven neurodegeneration, microglial activation could have an important role in modulating propagation of proteinopathies.

It is possible that, as the disease advances, microglia become dysmorphic and dysfunctional, resulting in amyloid accumulation due to failure of clearance and the secretion of proinflammatory cytokines, promoting neurodegeneration. While it is possible to have a protective role for microglia in early stages of the disease, microglial activation in established AD is likely to have a deleterious effect. It has been shown that higher cortical microglial activation correlated with lower Mini-Mental State Examination scores and regional cerebral glucose metabolic rates in established AD,^33^ supporting the view that microglial activation drives neuronal dysfunction in later stages of the disease as in established AD with significant cognitive dysfunction.^12,26,34^ It has also been shown that levels of microglial activation increase with disease progression in established AD.^12,33^ Recently, it has been demonstrated that brain inflammation is associated with amyloid deposition in the majority of MCI cases due to AD.^35^

There are 2 different types of activation pattern of microglial cells, allowing the classification of the cells into 2 different phenotypes: proinflammatory (M1), or classically activated; and the protective (M2) phenotype, which is alternatively activated. The switch from the anti-inflammatory to proinflammatory state is a dynamic process with a significant influence from peripheral inflammation.^36^ Jimenez et al.^37^ demonstrated a distinctive shift from anti-inflammatory to proinflammatory phenotype of microglial activation in the hippocampus of aged rodents.^38^ As depicted in the data available from Dryad (supplemental figure 1A, doi.org/10.5061/dryad.6mp3r21), one could argue that in the early stages, the protective phenotype of the microglia is activated. While TSPO tracers are unable to differentiate between the anti-inflammatory and proinflammatory phenotype, positive correlation between the microglial activation and gray matter volume suggests that the influence of early microglial activation may be beneficial, even though longitudinal studies are necessary to confirm this. Of note, recent studies suggest astrocyte activation also occurs in the early stages of AD pathology.^39,40^ One could argue that both astrocyte and microglial activation go hand in hand in the early stages of the disease. Moreover, a recent trial of minocycline in patients with traumatic brain injury was able to show that while minocycline reduced chronic microglial activation, it also increased neurodegeneration, further suggesting that microglial activation might have a reparative effect.^41^

On an individual basis, we have been able to demonstrate that 35% of patients with MCI had significantly increased [^11^C]PBR28 binding compared to controls. Previously, it has been shown that significantly higher cortical [^11^C]PBR28 binding is present in patients with AD compared with controls, but conflicting results have been reported in MCI.^42–47^ The discrepancy in the findings between the studies may partly reflect the different methods used for the analysis, along with heterogeneity of the patients with MCI who were studied. In this study, we quantified regional cerebral [^11^C]PBR28 V_T_ using a metabolite-corrected arterial input, while most other studies have normalized regional tracer activity to that of the cerebellum. However, the cerebellum may exhibit microglial activation in AD and, therefore, provides a suboptimal reference region. In addition, levels of microglial activation are likely to differ across different regions in different individuals and so averaging regional uptake and reporting a group mean in a heterogeneous disease may underestimate the true prevalence of microglial activation. However, limitations from the use of [^11^C]PBR28 have to be acknowledged, and those might be responsible for the lack of group differences and the small number of “microglial-positive” patients. [^11^C]PBR28 shows a high test-retest variability in healthy controls (approximately 15%), and TSPO expression in the brain might be influenced by peripheral leucocyte counts.^48,49^ Moreover, free fraction of plasma tracer is another source of variability and the analytical method used might contribute to the lack of group difference. Our study population included both MAB and HAB and, as previously demonstrated by our group, the rs6971 single-nucleotide polymorphism of the *TSPO* gene, other than affecting [^11^C]PBR28 and other second-generation TSPO tracers binding, does not affect any others of the clinical, neuropsychological, and biomarker characteristics of an AD or MCI population; thus, the results of the genetic subgroups can be applied to the entire MCI population.^50^

Contrary to conventional wisdom, demonstration of correlation with gray matter volume and hippocampal volume is of great significance to our knowledge about microglial activation and future therapeutic strategies to influence microglial activation. Hippocampal volume in AD correlates with cognitive performance, with higher hippocampal volume associated with better performance in cognitive function. Moreover, hippocampal atrophy is a predictor of progression to AD in individuals with MCI.^51^ However, atrophy in the AD trajectory does not only affect the hippocampus, and several VBM studies have shown that MRI gray matter density is generally reduced in AD.^52^ A recent meta-analysis of VBM studies reports that gray matter atrophy in medial temporal lobe structures is a predictor of conversion in MCI.^53^ The pattern of atrophy in our amyloid-positive patients with MCI is consistent with the AD profile (reduced hippocampal and amygdala volumes and enlarged lateral ventricles) while amyloid-negative patients show preserved medial temporal lobe structures but enlarged ventricles compared to controls, which cannot be attributed to age.^54,55^

Recently, we have demonstrated that there are 2 peaks of microglial activation in the AD trajectory,^25^ and one could argue that the presence of microglial activation that is associated with preservation of brain volume could substantiate the presence of an early protective peak of microglial activation.^56^ If the hypothesis of differential activation of microglia is true, one could further hypothesize that agents promoting an anti-inflammatory phenotype of microglia may be beneficial in the early stages of the disease, while agents suppressing the proinflammatory phenotype may be beneficial in later stages of the disease, especially when dementia sets in. However, brain volume changes may not be solely driven by neuronal loss. Trials of active antiamyloid immunotherapy in AD have shown that treatment was able to induce reduction of gray matter volume, especially in responders, while improving cognition. It is hypothesized that volume loss in these patients could be attributable to reduction in interstitial or glial constituents, as well as removal of amyloid plaques.^57,58^ Therefore, preservation of brain volume associated with microglial activation in our study could not be simplistically attributed to neuronal cell numbers preservation.

While our findings suggest that microglial activation in the early phase of the disease is associated with higher hippocampal and gray matter volume and point toward a beneficial role, there is significant evidence to suggest that microglial activation in the later stage of the disease is associated with neuronal damage.^59^ It is very likely that microglial activation in different stages is associated with different functions, and as shown in data available from Dryad (supplemental figure 1B, doi.org/10.5061/dryad.6mp3r21), multiple factors could contribute to microglial activation, neuroinflammation, and neurodegeneration, with and without the presence of amyloid. These factors may very well be acting during the prodromal stages of the disease. An effective therapeutic strategy might require selective targeting of the different stages of microglial activation.

A limitation of this study is that we did not perform a partial volume correction for [^11^C]PBR28 PET, even though when defining ROIs, we used a probabilistic atlas that takes into account variations in individual brain structures. While this may have resulted in an underestimation of cortical V_T_, it is likely that positive values will only be augmented with partial volume correction. Another limitation is the younger control group used in this study; however, studies have demonstrated that cortical microglial activation is minimally affected by normal aging in humans and its presence represents a disease-specific process.^60^ The study population is limited to 37 patients with MCI, so the results of this pilot study will be strengthened by studies with larger populations and longitudinal series.

## Conclusion

In this study, we report that [^11^C]PBR28 V_T_ was associated with higher gray matter volume and higher hippocampal volume in patients with MCI. Based on the findings from this study, one could argue that microglial activation may not always be detrimental, and in the early stages of neurodegenerative diseases such as AD, it may have a beneficial role in preserving brain volume. While further longitudinal studies are essential to evaluate the causal relationship, these findings from our pilot study have important implications in developing therapeutic strategies modulating microglial activation in AD and other diseases.

## Glossary

AD: Alzheimer disease
BPM: biological parametric mapping
GWAS: genome-wide association studies
HAB: high-affinity binder
HC: healthy control
MAB: mixed-affinity binder
MCI: mild cognitive impairment
MNI: Montreal Neurological Institute
PBR: peripheral benzodiazepine receptor
ROI: region of interest
SPM: statistical parametric mapping
TSPO: 18-kDa translocator protein
VBM: voxel-based morphometry
V_T_: volume of distribution
